# Supplementary material for: Long-term mutual phase locking of picosecond pulse pairs generated by a semiconductor nanowire laser
Source: Nat Commun. 2017 May 23;8:15521. doi: 10.1038/ncomms15521 (PMC5457509; doi:10.1038/ncomms15521)
Supplement: Supplementary Information — Supplementary Figures and Supplementary Notes [file ncomms15521-s1.pdf]

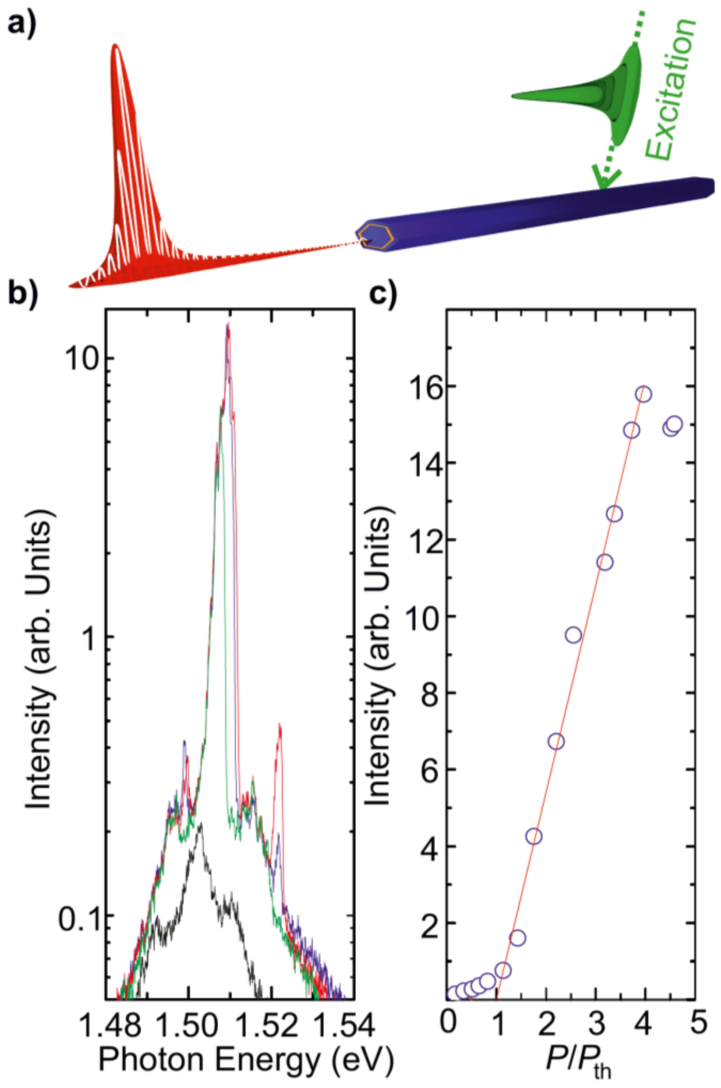

Supplementary Figure 1: Nanowire-laser output when excited using a single non-resonant pump pulse. a) Schematic image of a nanowire laser emitting a single pulse. b) Selected spectra recorded with different pump intensity P normalized to the threshold pump power,P_{th}, :$P/P_{\mathrm{th}} \sim$ 0.6 (black curve), $P/P_{\mathrm{th}} \sim$ 1.9 (green curve), $P/P_{\mathrm{th}} \sim$ 4.7 (blue curve) and $P/P_{th} \sim$ 5.3 (red curve). c) Characteristic Light-in/Light-out curve obtained from the nanowire-laser together with the linear fit (red curve) used to determine the lasing threshold.

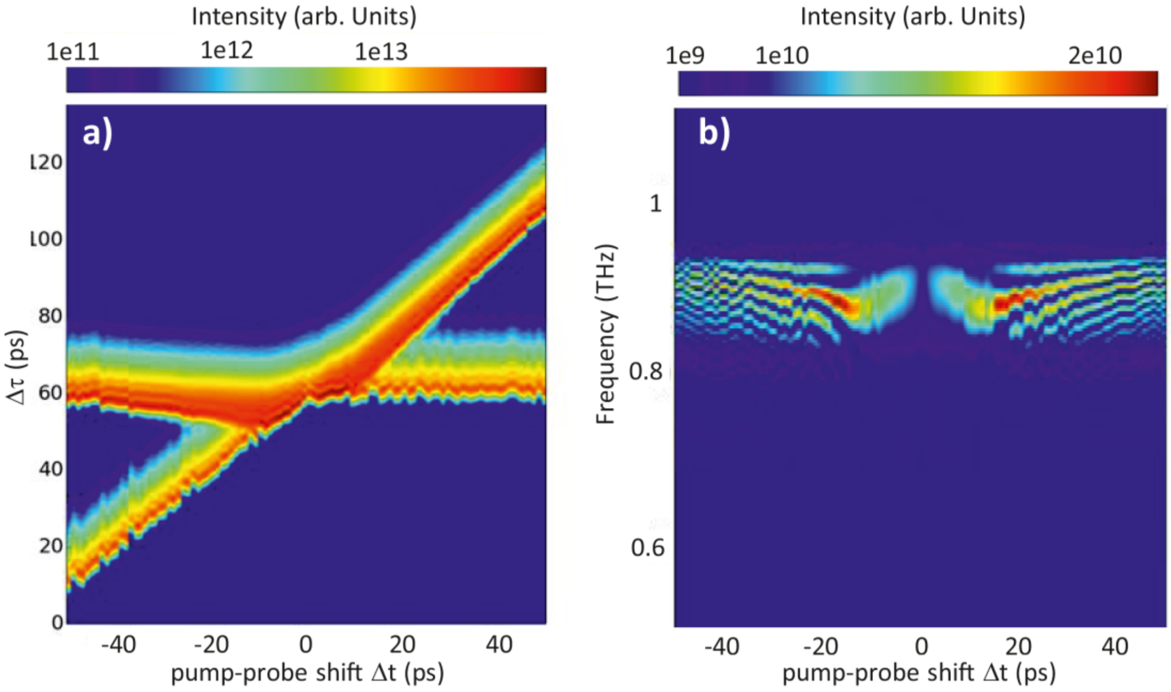

Supplementary Figure 2 – Impact of saturation of the reservoir state on the emission (a)Simulation showing intensity data in the time domain for $P_{probe} = P_{pump} = 4.7P_{th}$ including reservoir saturation effects (b) Discrete Fourier transform of the time-domain data showing weaker emission in the region close to ± 10ps corresponding to saturation of the reservoir due to Pauli blocking

Supplementary Note 1: Effect of Pauli-blocking in the reservoir

The input-output characteristics of the nanowire laser when subject to excitation by a single pump pulse were investigated and are presented in Supplementary Fig. 1. Note the saturation of the output intensity for $P_{\mathrm{pump}} \geq 4P_{\mathrm{th}}$ arising from Pauli blocking in the reservoir state into which the system is pumped. In the simulation this can be implemented by blocking the carrier injection into the reserevoir as soon as a maximum density is reached. We find that this Pauli blocking in the reservoir slightly changes the nanowire emission for small pump-probe delay

times, as presented in Supplementary Fig.2 in the time domain (a) and in the frequency domain (b) for a maximum occupation of $N_2 = 5$. This is in agreement with the experimentally observed data shown in the main part of the paper in Fig.1.

Supplementary Note 2: Assigning the temporal ordering of pulses in the Fourier transformed data in Fig 3.

In Fourier transforming the frequency-domain data (Fig. 1) into the time-domain (Fig. 3) one has to be careful about the assignment of the temporal ordering of the two emitted laser pulses. This is due to the fact that only positive frequency components are present that fix a relative time but not the absolute time scale. However, the assignment of the emission attributed to pump and probe excitation pulses can be linked with pulse-1 and pulse-2 in the emission as follows. In the experimental data presented in Fig. 3a positive (negative) Δt corresponds to the weak probe pulse arriving at the sample after (before) the more powerful pump pulse (defined by the experiment). When the probe pulse has insufficient power to induce lasing in the nanowire, the emission it induces is primarily spontaneous and incoherent. As such, it does not have a fixed phase relationship to the subsequently emitted laser pulse induced by the time delayed stronger pump pulse and, thereby, no interference is observed in the time integrated emission spectrum. In the Fourier transform of Fig. 3a, this corresponds to temporal components close to zero-time on the $\Delta\tau$-axis as seen at negative Δt in Fig. 3a. In contrast, for positive Δt the powerful pump that arrives first at the sample does induce lasing; it fixes the phase and the delayed "probe" also produces lasing with a fixed relative phase due to the pre-excitation. This results in interference in the time-integrated emission spectrum, leading to the feature observed away from the zero-time axis of the emitted laser pulse. The probe, therefore, gives rise to the feature labelled pulse-2 in the Fourier transformed data as seen in the figure, appearing only for positive Δt, i.e. when the powerful pump excites the sample before the probe. Note, the plot becomes symmetrical when the power of the probe pulse is increased into the lasing regime as well, since both pump and probe pulses induce lasing, fix the phase coherence and give rise to interference in the time integrated spectrum. Therefore, we can unambiguously associate pulse-1 as being emitted first and pulse-2 second for positive Δt, defining the positive time $\Delta\tau$ axis in Fig. 3. Note, the intensity of pulse-2 feature in Fig. 3 reflects only the modulation amplitude of the interference (see Figs. 1 & 2) and is not interpreted by our experiment.

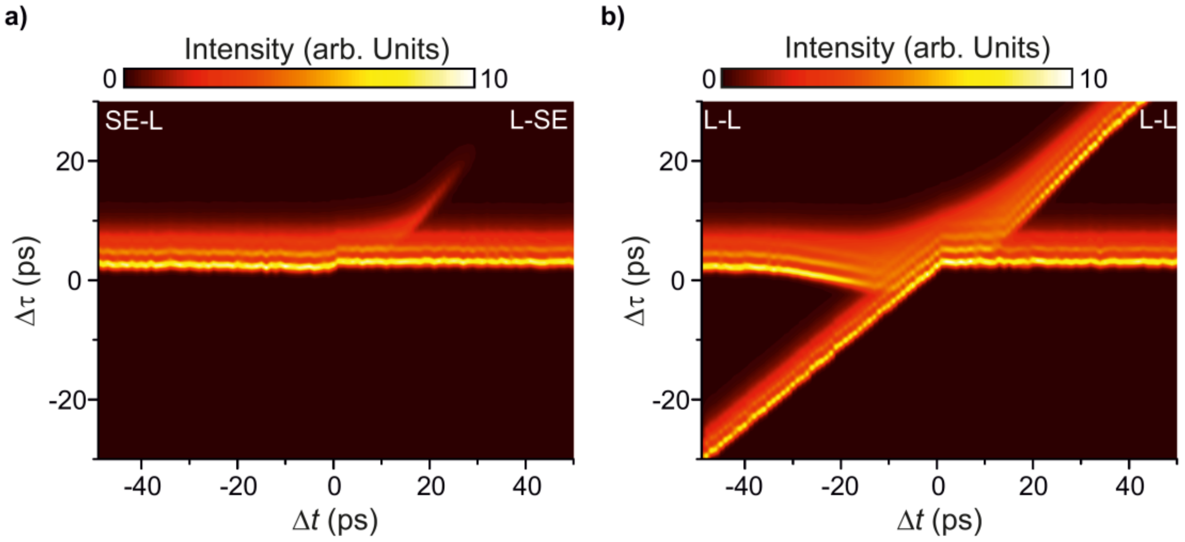

Supplementary Figure 3 - Direct simulations of the time dependent emission of the NW laser as a function of the pump-probe delay time Δt. (a) pump pulse in the lasing regime and the probe pulse in the SE regime (L-SE). (b) both, pump and probe pulse in the lasing regime (L-L).

To more firmly establish this assertion, Supplementary Fig. 3 shows the direct time traces obtained from our simulations for two cases; The first case occurs when P_{pump} is in the lasing regime and P_{probe} is weaker, in the spontaneous emission regime (denoted SE-L or L-SE depending on whether the weaker pulse arrives first at the system or vice-versa – (a)). The second case demonstrates the lasing-lasing (L-L – (b)) regime where both pulses are sufficiently strong to produce lasing. Here, the *y*-axis extends over positive and negative times. In Supplementary Fig. 3(a) we observe a strong emission pulse close to zero-time delay (horizontal line on Supplemetary Fig. 3) arising from the pump-pulse, as discussed above, and a weaker emission pulse for positive Δt due to emission induced by the probe pulse only after the system has previously been pumped. In contrast, for the L-L case we observe strong emission induced by both pump and probe pulses. In our experiments, we can interpret the double Fourier transformed data in Fig. 3 on the basis of these plots, and the "positive only y-axis" presented in the discrete Fourier transformed data of Fig. 3 corresponds to taking the absolute value of the y-axis of Supplementary Fig. 3 producing results similar to the L-L modeling curve in Fig. 3b. Thus, we are sure about the labeling of the emitted pulses in Fig. 3 from the evolution of the form of the data with P_{pump} and P_{probe}, both in experiment and theory.
